# Acute dacryocystitis in children with mononucleosis

**DOI:** 10.5935/0004-2749.2025-0044

**Published:** 2025-04-04

**Authors:** Silvana Artioli Schellini, Tammy Hentona Osaki

**Affiliations:** 1 Departamento de Especialidades Cirúrgicas e Anestesiologia, Faculdade de Medicina de Botucatu, Universidade Estadual Paulista “Júlio de Mesquita Filho”, Botucatu, SP, Brazil; 2 Departamento de Oftalmologia, Escola Paulista de Medicina, Universidade Federal de São Paulo, São Paulo, SP, Brazil

Dear Editor,

Congenital nasolacrimal duct obstruction (CNLDO) is a common cause of epiphora among children, and it occurs within the first weeks of life^(1)^. This condition is not rare, as it is influenced by anatomical variations in the lacrimal duct, lacrimal sac enlargement, syndromic associations, and infections^(2^,^3)^. Acute dacryocystitis is uncommon in children, generally occurring as a complication of CNLDO^(3)^.

Recently, an atypical case of a 4-year-old girl with no previous tearing who presented with unilateral acute dacryocystitis and pericystitis was reported. She had fever that started 5 days previously, marked cervical lymphadenopathy and hepatosplenomegaly, elevated C-reactive protein level, elevated white blood cell count, elevated liver function enzyme levels, and positive result for Epstein–Barr virus (EBV) on serology. She was diagnosed with infectious mononucleosis and started on intravenous ceftriaxone but with no improvement. After 3 days, a computed tomography (CT) scan revealed a collection within the lacrimal sac. Nasal endoscopy showed normal findings. The acute dacryocystitis was probably secondary to the infiltration of the nasola-crimal duct (NLD), causing luminal inflammation and narrowing of the NLD, with subsequent obstruction. Probing confirmed this hypothesis, detecting some resistance upon entering the NLD. After probing, intravenous ceftriaxone was continued for 48 h, and a 7-day course of oral amoxicillin/clavulanic acid was administered, which resolved the problem^(4)^.

This case highlights the following important and unusual aspects of NLD obstruction in children:

1. Epiphora started in a 4-year-old child, whereas CNLDO-related tearing started in the first weeks of life.
2. An association between epiphora and EBV features was confirmed via laboratory examination.
3. EBV-induced dacryocystitis is a possibility in hyperendemic areas for *Sporothrix schenckii*^(5)^.
4. The child had widespread systemic infectious mononucleosis and developed acute dacryocystitis and pericystitis, likely due to virus migration from the oropharyngeal mucosal epithelium to the deeper nasal lymphoid tissue, resulting in NLD inflammation and obstruction^(4)^.
5. Systemic cephalosporins did not improve the patient’s condition, and the CT scan revealed a lacri-mal sac abscess, requiring probing to prevent orbital cellulitis, cavernous sinus thrombosis, meningitis, brain abscess, and sepsis^(3)^.

Systemic antibiotic therapy is mandatory for at least 24 h before probing to reduce the risk of probinginduced bacteremia, which is a possibility in 4%–33% of cases^(3)^.

Nasal endoscopy and probing are successful in most cases of acute dacryocystitis unresolved by systemic antibiotics alone. Endoscopic guidance during probing is extremely useful for diagnosing associated intranasal cysts, complex CNLDO, endoscopic drainage, and collecting additional purulent material for microbiological examination^(1^,^2)^.
